# Rest-Activity Rhythm Patterns and Physical Functional Performance in Community-Dwelling Older Men

**DOI:** 10.1093/geroni/igab046.1305

**Published:** 2021-12-17

**Authors:** Dorothy Chen, Gregory Tranah, Patrick Bradshaw, Terri Blackwell-Hoge, Jamie Zeitzer, Sonia Ancoli-Israel, Kristine Yaffe, Katie Stone

**Affiliations:** 1 Sutter Health, SAN FRANCISCO, California, United States; 2 Sutter Health, San Francisco, California, United States; 3 UC Berkeley, Berkeley, California, United States; 4 Stanford University, Palo Alto, California, United States; 5 UC San Diego, La Jolla, California, United States; 6 UCSF, UCSF, California, United States; 7 University of California, Berkley, San Francisco, California, United States

## Abstract

Sleep and activity patterns have been linked to physical performance in older adults. Traditional parametric models of 24-hour activity rhythms fail to adequately capture specific diurnal sleep and wake patterns; functional principal components analysis (fPCA) is a non-parametric approach that addresses this limitation. Using fPCA, we modeled accelerometry data from n = 2,960 participants in the Osteoporotic Fractures in Men (MrOS) ancillary sleep study (mean age 77y) and examined cross-sectional associations with gait speed and grip strength measurements. Lower daytime activity (expected difference = -0.049 [-0.072, -0.028] m/s), increased sleep duration and a reduced midday dip in activity (-0.015 [-0.035, 0.006] m/s) were modestly associated with worsening gait speed. A modest association between both later sleep and wake times and increased sleep duration with worsening grip strength outcomes was observed (-1.11 [-1.90, -0.32] kg). Specific daily activity patterns may serve as predictive biomarkers for changing physical function in aging populations.

